# Tagging Emissions from Indoor Biomass Combustion with a Cost-Effective Sensor Array: From Design to Field Deployment in Rural Indian Households

**DOI:** 10.1021/acs.est.4c08533

**Published:** 2025-07-17

**Authors:** Nguyen Thanh Duc, Daniel Montecinos, Jay Prakash Kumar, Sayantan Sarkar, Roshan Wathore, Johannes Felix Amann, Julian Joppich, Sumedha Lawande, Rajesh Kumar Ranjan, Joyanto Routh

**Affiliations:** † Department of Thematic Studies-Environmental Change, 4566 Linköping University, Linköping 58183, Sweden; ‡ Department of Environmental Science, 206411 Central University of South Bihar, Gaya 824236, India; § School of Civil and Environmental Engineering, 231997 Indian Institute of Technology, Mandi 175005, India; ∥ Academy of Scientific and Innovative Research (AcSIR), Ghaziabad 201002, India; ⊥ 29581 CSIR-National Environmental Engineering Research Institute, CSIR-NEERI, Nagpur 440020, India; # Lab for Measurement Technology, 9379 Saarland University, Campus A5 1, 66123 Saarbrücken, Germany

**Keywords:** biomass combustion, CO_2_ emissions, cost-effective, sensor array, BME688, VOC fingerprint

## Abstract

In this study, we introduce an innovative method for monitoring emissions from indoor biomass combustion, a prevalent practice in rural households in the Indo-Gangetic Plains. Our approach utilizes a portable and cost-efficient sensor array with advanced data handling, employing commercially available sensors to measure CO_2_, CO, NO_2_, SO_2_, PMs, VOC, NOx, cookstove and ambient temperature, relative humidity, and pressure. We developed hardware and software to gather and process sensor data and control the temperature cycle using the BME688 sensors. The field deployments reveal that CO_2_ emission from a cooking event is ∼2.3 ± 1.5 kg CO_2_ per family. Extrapolating this data, the total emissions from biomass (e.g., fuelwood, crop residues, animal dung, and charcoal) for household cooking in rural areas of India are estimated to be around 0.6 ± 0.4 teragrams (Tg) of CO_2_ per day. The integration of dual BME688 sensors, leveraging the standard Bosch Software Environmental Cluster library and temperature cycling, achieves an impressive 95% accuracy in fingerprinting emissions from different fuel types. This capability enables the creation of a comprehensive database, where each CO_2_ emission data point is meticulously linked to the original biomass source. This level of real-time detail, previously unattainable, greatly enhances our ability for emission quantification and offers broad applicability for mitigation efforts.

## Graphical abstract

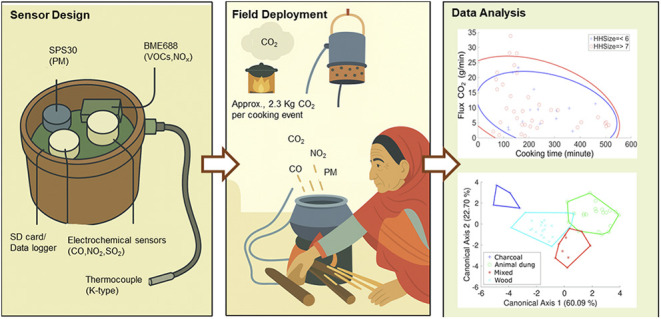

## Introduction

The combustion of biomass, such as fuelwood, crop residues, animal dung, and charcoal, is still a ubiquitous and cheap source of energy for cooking in many rural households worldwide, accounting for nearly 36% of the world’s population.^1,2^ Biomass burning for cooking or heating is deeply rooted in tradition. It remains a practical necessity for low-income households, mainly because of its widespread availability and minimal or no cost. However, biomass burning releases a complex mixture of particulate matter (PM), carbon dioxide (CO_2_), carbon monoxide (CO), nitrogen dioxide (NO_2_), sulfur dioxide (SO_2_), volatile organic compounds (VOC), and other toxic gases. The cumulative effects of these emissions have been associated with respiratory diseases, cardiovascular problems, and other adverse environmental impacts.^3,4^ Recent studies have provided well-documented evidence indicating the severe environmental and public health costs of biomass burning.^5–9^ To mitigate the economic and health impacts, studies highlight that the lack of in situ measurements leads to underestimating emissions and ineffective policies in regions with widespread biomass burning.^10–12^ Therefore, improving the inventories for fuel emissions is essential to accurately identify and quantify the specific sources contributing to local air quality. This will help reduce the uncertainties in emissions from different sources, improve air quality modeling predictions, and support targeted mitigation actions.^13^

Since the 1970s, research into air pollution from biomass burning has seen rapid advancements in science and technology for studying pyrogenic emissions.^14^ Earlier studies relied heavily on surveys and questionnaire-based evaluations, which assessed the environmental impacts of cookstove utilization, fuel type, and household conditions (e.g., ventilation, number of inhabitants, and cooking practices). These efforts have contributed to building a foundational knowledge base in this field. Conventional methods for monitoring air quality typically rely on costly reference instruments, ranging from commercial portable analyzers^15^ and advanced mass spectrometers^16,17^ to large-scale infrastructure, such as airborne infrared spectroscopy and high-resolution satellite observations.^18–21^ Despite the substantial insights into emission characteristics provided by the development of advanced methods and the efforts of many research groups, their application for high-resolution spatial and temporal monitoring of indoor biomass emissions in rural households remains limited. These constraints stem from prohibitive costs, the challenge of providing a comprehensive picture while accounting for different emission sources, the bulky design of reference-grade instruments, and the operational challenges in field settings where a reliable power supply is often lacking.

Emerging low-cost sensor technology provides real-time monitoring opportunities for robust air quality evaluation on a much larger geographical scale with much finer temporal and spatial resolution in both outdoor and indoor environments. Low-cost sensors offer a practical solution for comprehensive and rapid air quality monitoring and assessment. Consequently, the low-cost sensor applications in monitoring biomass burning have grown over the years.^22–26^ These studies, conducted in laboratory settings and rural households, reported monitoring stove use,^23^ temperature, humidity, PM,^18,19^ CO_2_, and CO^27^ using innovative devices designed to monitor biomass burning. However, despite the growing demand for such devices, advancements in sensor technology have yet to be widely shared in open-source publications. This limitation restricts their accessibility primarily to well-funded research groups or industries involved in their development, leaving smaller, resource-constrained research groups disadvantaged.

The development of BME688, a metal oxide semiconductor (MOS) sensor that can detect VOC, volatile sulfur compounds (VSC), and other gases such as CO and hydrogen, makes them ideal for monitoring emissions from biomass burning. This sensor can run a temperature cycled operation (TCO) to increase the sensitivity and selectivity for gas discrimination and quantification of target gases in a background of other interfering gases.^28^ Combined with advanced signal processing, this dynamic operation proves advantageous, enabling numerous indoor air quality (IAQ) monitoring studies to be conducted successfully.^25–28^ However, to our knowledge, the TCO on BME688 has not been applied to monitor indoor biomass burning, a practice widespread in the Global South that poses significant health and economic challenges.^6–9,12^ This gap hinders the effective monitoring of emissions and the development of informed policy directives.

This study addresses a critical challenge in public health and environmental monitoring, focusing on developing and implementing a sensor module designed explicitly to investigate emissions from biomass burning in rural areas, including various gases and PM. The research was conducted in India—a rapidly growing economy with a population of 1.4 billion, where >41% of households still rely on solid fuels for cooking and recognized as the world’s largest energy poverty hotspot.^29^ The study leverages the BME688 sensor, integrated with an array of additional sensors, to identify the unique chemical fingerprints of gases emitted from various fuel types commonly used in traditional cooking stoves in rural areas in the Indo-Gangetic plains. Employing cost-effective, factory-calibrated sensors supported by calibration documentation enables real-time and robust data collection for comprehensive IAQ monitoring and assessment. By combining scientific rigor with community engagement, this research enhances our understanding of the health and environmental impacts of biomass burning in resource-constrained settings. The insights generated will be crucial in informing policy directives to mitigate exposure risks from PM and toxic gases, aiming to improve IAQ and public health outcomes in impoverished rural households.

## Method

### Hardware and Software Design

The sensor node is custom-built to be deployed about 0.8–1.5 m above the cookstove, where heat and emission of soot, PM, and gases are high. The portable, lightweight device can endure heat, soot, and other gaseous emissions from cookstoves without impacting functionality. It is particularly well-suited for deployment in rural areas where the electric supply is often unstable and it can be used to measure indoor and outdoor ambient air quality. Additionally, it is cost-effective and straightforward, enabling researchers worldwide to configure this open-source solution easily. The sensors are selected from the low-cost commercially available electrochemical sensors, including NO_2_-A43F, CO-A4 (modified measurement range up to 500 ppm), SO_2_-A4 (modified to measure up to 100 ppm; Alphasense, U.K.), optical sensors: SPS30 for particulate matter (PM) measurement (Sensirion, Switzerland), Sunrise CO_2_ (Senseair AB, Sweden 006-0-0008); metal oxide sensors: two BME688 (Bosch) and SGP41 (Sensirion) for VOC and NOx measurements. A 2.5 m length K-type thermocouple (RS Pro 334-2622) coupled with an I2C Thermocouple Amplifier Module MCP9600 (Seeed Studio 101020594) measures the cookstove temperature.

These sensors are controlled, and the data is collected on a 32 GB Netac micro-SD memory card (NT02P500STN-016G-R) using an Arduino MKR Zero (ABX00012) board. For extended applications where telecommunication is available, data from this sensor node can be wirelessly sent out to a server using a radio communication module. The date and time (GMT) are accurately recorded and time-stamped using the MKR GPS shield (ASX00017) readout. The Arduino code is configured to utilize the I2C digital communication sensor in power-saving mode, ensuring extended operational time when powered by a 5 V power bank. The SD memory card has two data file types, including defined and nondefined parameters. The specified parameter refers to sensors monitoring PM, CO_2_, CO, NO_2_, SO_2_, VOC, NOx, and IAQ index. The NOx index reported by the SGP41 sensor is a dimensionless variable positively correlated with the concentration of nitrogen oxide (NO and NO_2_) in the environment. The IAQ index from the BME688 sensor is a dimensionless metric that is positively correlated with the concentration of indoor air pollutants, especially VOC. The index typically ranges from 0 (excellent air quality) to 500 (heavily polluted air). These indices do not directly measure specific gas concentrations (as in ppb or ppm range), but provide a processed signal for trend analysis and air quality classification. These electrochemical sensor data, which include working and auxiliary electrode voltage of NO_2_, CO, SO_2_, and raw resistance value of SGP41 and BME688 sensors, are read every 3 to 5 s for a minute, and statistical averages and standard deviations are generated. This reduces the amount of data that needs to be logged on the SD card while retaining essential information. These raw NO_2_, CO, and SO_2_ sensor voltages could be converted to concentrations using factory calibration factors for each sensor and corrected for temperature using the Application Note AAN 803-05 algorithm. However, due to the lack of a facility for postdeployment calibration versus reference equipment, the following gases, NO_2_, CO, and SO_2_, are treated as dimensionless variables for trend analysis and air quality classification. The CO_2_ and SPS30 sensors report concentrations using their respective operational software library provided by the vendor. The nondefined data parameters are gas resistance values of two BME688 sensors at two I2C addresses, 0x76 and 0x77. The BME688 at address 0x76 (called BME0x76) is run with the standard Bosch Sensortec Environmental Cluster (BSEC) library with generic_33v_3s_4d configuration in bsec_iaq.txt. The BSEC library is a software that provides advanced sensor data processing and fusion for the BME688 sensor. It enables the extraction of ambient temperature, humidity, pressure, and gas resistance from raw sensor data. The other BME688 at address 0x77 (BME0x77) is run using custom gas scanner functions. This function digitally sets the heater beneath the MOX film with the temperature cycles from 200 to 400 °C in 25 °C steps (Figure SI 1). The temperature range of 200 to 400 °C is not directly measured but is calculated based on the change in heater resistance within the circuit. Before each temperature increment is implemented, the sensor is programmed to be set to 400 °C to refresh the sensor surface. This process involves heating the MOX layer to a high temperature, which helps to desorb any adsorbed gases and contaminants from the sensor surface and effectively “clean” it. High temperatures increase the volatility of adsorbed substances, facilitating their release from the sensor surface. This process allows the sensor to be reactivated while preserving its sensitivity and overall functionality for subsequent measurements.^30,31^ The BME688 sensor is heated in forced mode for 700 ms, with the cycle repeated five times at each temperature level. The temperature range was chosen to ensure the comprehensive detection of VOC and VOS emissions during biomass burning. The new data files are created daily; if a data file crashes, a new version is made on that date. The board design and embedded code are available at: https://gitlab.liu.se/thang92/tagging-biomass-combustion/-/tree/main/ArduinoMKR_Zero_AirQuaTag_01_code.

The sensor housing is made from a polypropylene (PP) plastic drainage pipe sliding sleeve (110 mm ID) and a sewage pipe end stop (110 mm ID). These components are chosen for their durability and waterproof capacity, which safeguards the electronic components. They are also inexpensive and readily available at any hardware store. The sewage pipe end stop is cut to fit with the electronic carrier board (Figure SI 2). The electronic carrier board is designed to carry 5 and 3.3 V sensors and supplement modules. A USB-A plug feeds this board with a 5 V DC 2A phone charger or power bank. The 5 V DC is stepped down to 3.3 V DC using one of three commonly available downregulated voltage chips (REG1117F-3.3 V, LM2937-3.3 V, or TC1264-3.3 V). This allows the possibility of this carrier board working with both 3.3 and 5 V sensors. In addition, the board has additional sockets to connect to commercial I2C communication sensors. This board is also designed to carry an analog front end (AFE) board for three electrochemical sensors and one photoionization detector VOC sensor or four electrochemica sensors A4 for air quality sensors (Alphasense). The analog output signals are converted to digital signals using three ADS1115 16-bit ADC boards (Adafruit). The total material cost is about 700 USD, and the board design is available at https://gitlab.liu.se/thang92/tagging-biomass-combustion//tree/main/ElectronicBoard_design.

### Field Deployment

The system was developed and assembled at Linköping University, Sweden, and evaluated in our laboratory for its functionality in different microenvironments. In the laboratory, the software of the sensor node was optimized to ensure the sensor node could continuously operate even under challenging conditions such as power cuts. The CO_2_ sensor response was evaluated using a Los Gatos Research (LGR) instrument under varying temperature and relative humidity (RH) (Figure SI 3). A prototype was also sent to the National Environmental Engineering Research Institute (NEERI) in Nagpur, India, to examine whether the sensors’ measurement range could be exceeded and assess their durability under real-world conditions in the Cookstove and Emission Testing Laboratory (Figure SI 4). After preliminary testing, 12 units were deployed in 36 households across two states, including Bihar and Jharkhand, as depicted in Figure SI 5; they were selected based on the fuel type used for cooking, including animal dung, wood, crop residues (used alone or in various proportions with different biomass sources), and charcoal. The sensor array was hung or placed about 1–1.5 m above the cookstove, depending on the dimensions of the kitchen (Figure 1). The measurement duration in each household varied from 24 to 36 h, aiming to capture at least two cooking events and one background measurement. A set of size-segregated aerosol samples was collected using a 5-stage cascade impactors (Sioutas, SKC Inc., USA) operated at a flow rate of 9 L min^–1^ (Leland Legacy Pump, SKC Inc., USA). The sample collection duration matched individual cooking cycles. Samples were collected on prebaked quartz microfiber (QMA) filters (Whatman, U.K.) of 25- and 37 mm diameters in the size ranges of 2.5–10, 1–2.5, 0.5–1, 0.25–0.5, and <0.25 μm. The filters were transported to the laboratory in sealed polypropylene (PP) Petri dishes and stored at −4 °C until analysis.

**Figure 1:**
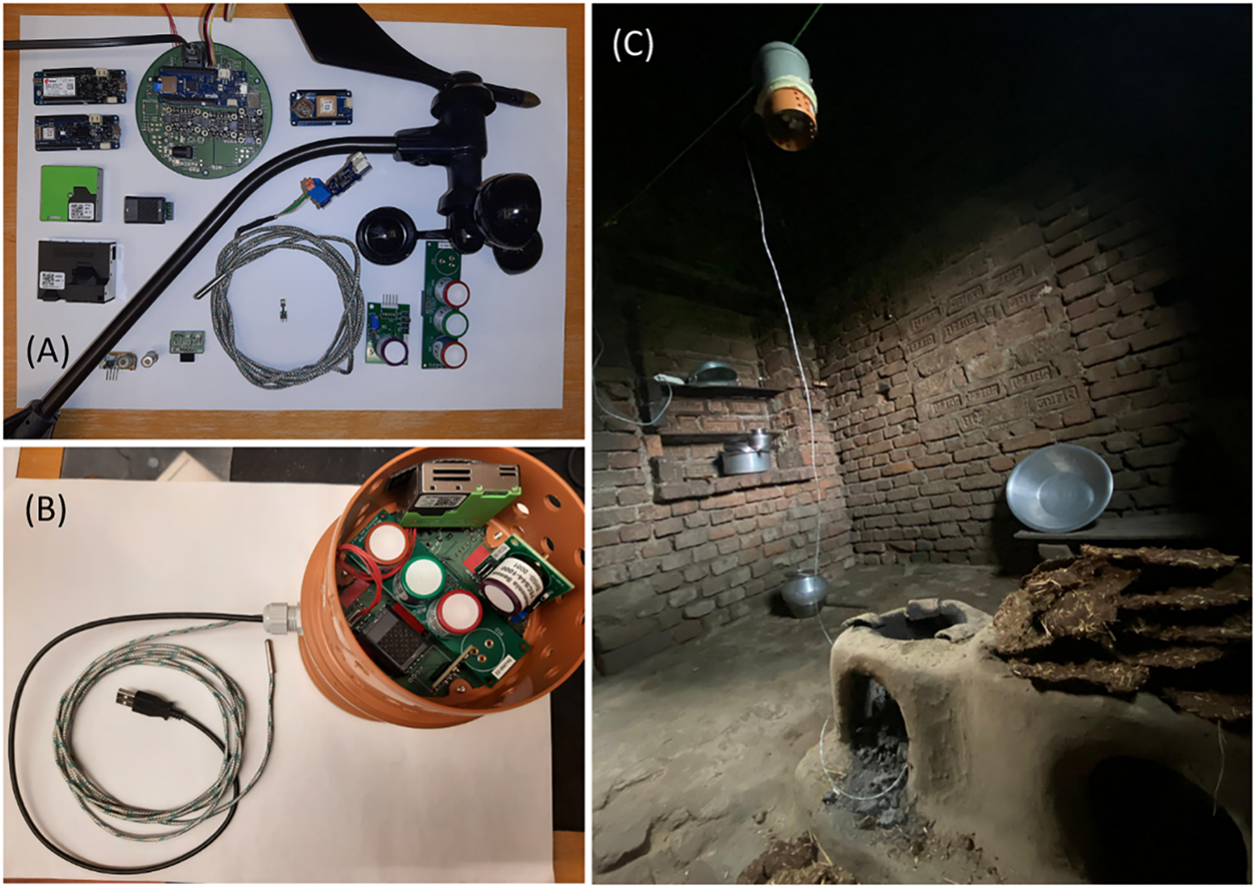
(A) Modular design array, (B) placement of sensors inside a drainage pipe for all-weather protection, and (C) deployment of sensors above a traditional indoor cookstove to monitor emissions during a cooking event. A K-type thermocouple is inserted into the cookstove to measure the temperature.

After completing the measurements, the data was transferred to the MATLAB cloud drive, and the SD cards were reformatted to ensure they were ready for the next deployment cycle. Following this, a cross-sensor comparison was carried out to check the functionality of sensor nodes. The sensor nodes were placed inside an airtight plastic glovebag (Figure SI 6) and flushed with medical oxygen for 1–2 h. All the sensors were exposed and measured in the same clean and humid air, and the data were used to assess signal drift or deviations between individual sensor nodes.

### Data Handling

The data is processed using MATLAB with the open-source software MatStats and DA3V from GitHub.^32–34^ The data files with different dates were merged before extracting the cooking events and background air quality. K-mean clustering (with *k* = 2) was applied to the data set for the dominant low values of MassPM_10_ and stove temperature. Cooking time was determined by abrupt changes in smoke and stove temperature when their measurement is higher than 1.5 times the dominant low values (e.g., MassPM_10_ > 100 μg/m^3^ and stove temperature >100 °C). Smoke was occasionally detected before the cookstove temperature increased. This was mostly due to delayed heating or the use of combustible materials such as paper, kerosene, twigs, or burning cinders to aid ignition. Therefore, smoke was detected in many cases before the cookstove temperature increased. The background condition was assessed when there was no smoke (e.g., MassPM_10_ < 100 μg/m^3^), and the stove temperature was low (<60 °C). All parameters, including minimum, maximum, average, standard deviation, and total gas emissions (peak area under the data line), were extracted to classify and quantify an event extending from cooking to its conclusion (before reverting to the background state). The sensor provided data on stove temperature, mass concentration (μg/m^3^) of PM_1_, PM_2.5_, PM_4_, PM_10_, and number concentration (#/m^3^) of PM_0.5_, PM_1_, PM_2.5_, PM_4_, PM_10_, a high precision typical PM size (μm), NOx index, total VOC, CO_2_, NO_2_, CO, SO_2_ concentrations, IAQ, and atmospheric conditions of temperature, relative humidity (RH), pressure, and gas resistance values from the standard BSEC library and heating cycle. The background parameters were subtracted from the cooking parameters to quantify source emissions. The background period was defined as the interval before and after the cooking events, characterized by low stove temperature and PM concentrations, ensuring that the measurements accurately reflect the ambient conditions without the influence of cooking activities.

The CO_2_ flux is calculated based on the upward movement of the hot air from the cookstove to the sensor node and the CO_2_ concentration measured at the sensor node. The lower density of hot air drives the upward velocity of buoyant flow compared to the ambient air. It is calculated from the temperature in the cookstove and ambient atmospheric conditions, including temperature, RH, and pressure^35^ as follows:

1 $$u=\sqrt{2\times g\times h\times \frac{\left(\rho_{\mathrm{ambient}}-\rho_{\mathrm{hot}\_\mathrm{gas}}\right)}{\rho_{\mathrm{ambient}}}}$$

where *u* is the upward velocity (m/s), *g* is the gravitational acceleration (9.81 m/s^2^), *h* is the height between the cookstove and the sensor node (m), ρ_ambient_ is the ambient air density, which is correlated with ambient temperature, RH, and pressure, and ρ_hot_gas_ is the density of the hot gas plume associated with the cookstove temperature.

The ambient air density can be calculated as follows:

2 $$\rho_{ambient}=\frac{P_{atm}}{R_{dry\;air}\times T_{atm}}\left(1-\frac{R_{water\;vapor}}{R_{dry\;air}}\times \frac{P_{water\;vapor}}{P_{atmosphere}}\right)$$

where *P*_atm_ is ambient atmospheric pressure (Pa) measured by the BME688 sensor, *T*_atm_ is ambient temperature (K), *R*_dry air_ is the gas constant for dry air (287.05 J/kg.K), *R*_water_vapor_ is the gas constant for water vapor (461.5 J/kg.K). *P*_water vapor_ is the partial pressure of water vapor (Pa), calculated with the Magnus-Tetens formula as follows:

3 $$P_{\mathrm{water}\;\mathrm{vapor}}=\mathrm{RH}\times 610.78\times e^{\left(17.27\left(T_{\mathrm{atm}}-273.15\right)/T_{\mathrm{atm}}-35.85\right)}$$

The density of the hot gas plume can be approximated using the ideal gas law at the respective cookstove temperature as follows:

4 $$\rho_{\mathrm{hot}\_\mathrm{gas}}=\frac{P_{\mathrm{atm}}}{R_{\mathrm{dry}\;\mathrm{air}}\times T_{\mathrm{cookstove}}}\left(1-\frac{R_{\mathrm{water}\;\mathrm{vapor}}}{R_{\mathrm{dry}\;\mathrm{air}}}\times \frac{P_{\mathrm{water}\;\mathrm{vapor}}}{P_{\mathrm{atmosphere}}}\right)$$

This hot air carries the combustion products through the stove and passes the sensor node, where the CO_2_ gas concentration is measured to generate the CO_2_ gas flux as follows:

5 $$F_{{\mathrm{CO}}_{2}}=\rho_{\mathrm{hot}\;\mathrm{gas}}\times u\times 60\times A\times C_{{\mathrm{CO}}_{2}}$$

where *F*_CO2_ is CO_2_ flux (kg/min), A is the cross-sectional area of the cookstove (footprint area m^2^), and the typical radius is about 0.2 m. *C*_CO2_ is CO_2_ concentration (ppm) converted to a CO_2_ mass fraction as follows:

6 $$C_{{\mathrm{CO}}_{2}}\left(\mathrm{kg}/\mathrm{kg}\right)=\frac{{\mathrm{CO}}_{2\left(\mathrm{cookevent}\right)}-{\mathrm{CO}}_{2\left(\mathrm{noncookevent}\right)}}{10^{6}}\times \frac{M_{\mathrm{CO}2}}{M_{\mathrm{air}}}$$

where *M*_CO2_ is the molar mass of CO_2_ (44.01 g/mol), *M*_air_ is the molar mass of dry air (28.97 g/mol).

The total CO_2_ emission is calculated as follows:

7 $${\mathrm{CO}}_{2\left(\mathrm{cook}\_\mathrm{emission}\right)}=\int_{\mathrm{start}\;\mathrm{cook}}^{\mathrm{end}\;\mathrm{cook}}F_{\mathrm{CO}2}\times {dt}_{\mathrm{cook}}$$

where CO_2(cook_emission)_ (kg) – emitted CO_2_ from the cooking event, d*t*_cook_ – cooking time (min).

The classification of fuels was tested using nondefined parameters from the BME688 gas resistance values. Because the BME688 sensor measures VOC and VOS, only the first five measurements for 10 min are used to avoid measuring VOC emitted while cooking food. For each temperature segment of the cycle, gas resistance values were used to calculate the following parameters, including the average slope for gas resistance, median, and standard deviation (indicating variation within each row), range (difference between maximum and minimum values), interquartile range (IQR) to assess statistical spread, skewness and kurtosis to characterize the distribution shape, and the Fast Fourier Transform (FFT) for frequency-domain analysis. All these parameters were calculated using MATLAB’s built-in functions.

The features from the BME sensors are extracted using the following equations for a single temperature (i) as follows:

- Average Slope: ${\mathrm{avgSlo}}_{i}=\frac{1}{5}\sum_{j=1}^{5}\mathrm{polyfit}\left(1:5,{{\mathrm{resistance}}_{\mathrm{data}}}_{i},1\right)\left(1\right)$

- Median: *median*_*i*_ = *median*(*resistance_data*_*i*_)
- Standard Deviation: *std*_*i*_ = *std*(*resistance_data*_*i*_)
- Range: *range*_*i*_ = *range*(*resistance*_*data*_*i*_)
- Interquartile Range: *iqr*_*i*_ = *iqr*(*resistance*_*data*_*i*_)
- Skewness: *ske*_*i*_ = *skewness*(*resistance_data*_*i*_)
- Kurtosis: *kur*_*i*_ = *kurtosis*(*resistance_data*_*i*_)
- FFT Energy: ${\mathrm{fft}}_{i}=\frac{\sum {\left|\mathrm{fft}\left({\mathrm{resistance}\_\mathrm{data}}_{i}\right)\right|}^{2}}{\mathrm{numel}\left({\mathrm{resistance}\_\mathrm{data}}_{i}\right)}$

The feature matrix for BME0x77 of a cooking event can be represented as in (eq SI 1). A similar feature matrix is generated for BME0x76 using resistance data recorded in parallel with BME0x77. The whole assessment derives 142 statistical features for each cooking event. These features are used in Linear Discrimination Analysis (LDA) to investigate the possibility of classifying different cooking fuels based on their emission characteristics.

Cross-sensor comparison is performed after flushing the sensors with medical oxygen (2 L per minute for about 2 h), ensuring that CO_2_ concentrations are low for baseline consistency. This marks the beginning of extracting the zero-measurement data. When oxygen flushing stops, ambient air gradually enters the plastic glovebag. The statistical parameters of each sensor, such as mean, median, range (maximum-minimum), and the slope of the change over time, are calculated to analyze the sensor’s performance and data trends. These metrics provide insights into the central tendency, variability, and rate of change in the sensor’s measurements, enabling a comprehensive assessment of the observed phenomena. The calculated parameters for all sensor nodes are compared, and extreme outliers are identified using the interquartile range. A sensor must be replaced if it exhibits an outlier in all four parameters, mean, median, range, and slope. If a sensor has an outlier only in the mean and median but not in the range and slope, it suggests a baseline drift in the sensor’s measurement. Additionally, if the sensor consistently reports zero values, this likely indicates a faulty connection, warranting a physical inspection of the connector for potential functional issues. A MATLAB script was developed to perform this check and report the output as a heat map to detect which sensor has a problem or is malfunctioning. This heat map visually highlights potential issues, such as outliers, baseline drift, or bad connections, enabling quick identification and troubleshooting of malfunctioning sensors (Figure SI 7). The MATLAB scripts used for data processing are available at: https://gitlab.liu.se/thang92/tagging-biomass-combustion/-/tree/main/Matlab_dataprocessing_code (10.5281/zenodo.13120952).

## Results

Due to random power cuts during the cooking events, data is missing from 8 out of 36 household deployments. From the logged data files, 57 cooking events were identified for different fuel types: animal dung, wood, mixed fuel (animal dung mixed with straw, crop residue, etc.), and charcoal. The data was further categorized to indicate the household status in terms of kitchen ventilation (i.e., open, semiopen, and closed units) and family size (≤6 people or >7 people/household).^36,37^ Open units refer to kitchens with no walls or barriers, allowing unrestricted airflow. Semiopen units have partial barriers, such as half-walls or large windows, providing some ventilation but not as much as open units. Closed units are fully enclosed kitchens with minimal ventilation. This categorization is based on assessments conducted at the field site.

The data indicated that the CO and SO_2_ sensors reached their maximum measurement range of around 6 ppm just 3 min after the fire started, even though the AFE board was modified to support readings up to 500 ppm. Therefore, these sensors are only provide insights into the background environment when no cooking activity occurs. The SGP41 for VOC and NOx only reported the raw values but did not report the NOx index (only 0 and 1 values) after temperature and RH corrections; the VOC index takes a long time to stabilize (up to a few hours). This was probably due to the frequent power cut-offs disrupting the stabilization process and insufficient operation time (<24 h) to establish the NOx relative index. The other sensors worked well during the cooking events and background measurements.

The sensors are intended to be consistently installed in the “breathing zone” (about 1.2–1.8 m from the floor) to ensure accurate air quality measurement at the height most relevant to human exposure. However, due to practical constraints such as low roof height, this is not always feasible; as a result, the sensor array is typically deployed at a height of 0.8–1.5 m. At these heights, the maximum temperature and RH at the sensor node above the cookstove are 39.1 ± 5.7 °C (28.9 to 54.2 °C) and 73.4 ± 5.9% (27.2 to 83.8%), respectively. The correlation tests showed no significant relationship between the height of the sensor node relative to the cookstove and either the temperature at the sensor node (*R* = 0.193, ρ-value = 0.15) or the PM_10_ concentration (*R* = 0.250, ρ-value = 0.06). This suggests that cookstove emissions rise and disperse in a manner largely unaffected by the sensor node height.

During the cooking event, the thermocouple recorded a maximum stove temperature ranging from 261 to 1266 °C and mass PM_10_ from 1.0 to 1.42 × 10^5^ μg/m^3^, indicating the high variability in emissions depends on cooking conditions and fuel type. In environments with elevated PM levels, the SPS30 sensor is programmed to accelerate the fan to a maximum speed for 10 s to blow out the accumulated dust whenever the sensor node is turned on. The PM sensor measured for 10 s and before entering an idle state for 50 s. This cycle helps conserve power while maintaining precision in generating PM emissions data. The results showed higher particulate concentrations during cooking than those observed during noncooking periods. The CO_2_ optical sensor operated in periodic mode, measuring for 4 s and then idling for the remaining 56 s of each minute. The CO_2_ concentrations ranged from 416 to 4346 ppm, effectively capturing the variability between noncooking and cooking activities. The integrated peak area from high CO_2_ concentrations during cooking revealed that CO_2_ emissions during the cooking event were positively correlated with the cooking time (*R* = 0.6838, ρ-value = 2.83 × 10^–8^; Figure 2). The N-way ANOVA analysis examining the relationship between CO_2_ emission and household family size returned a ρ-value of 0.055, indicating no statistically significant difference in CO_2_ emissions between households with larger or smaller family sizes. The larger family size tends to have higher CO_2_ flux but shorter cooking time. The low CO_2_ flux with longer cooking time can be observed at both family sizes. However, uncovering the nuances of different kitchen types, stove-use habits, types of food cooked, and other influencing parameters through sensor data will require considerable time and effort for detailed evaluation to fully understand the variability in emissions.

**Figure 2:**
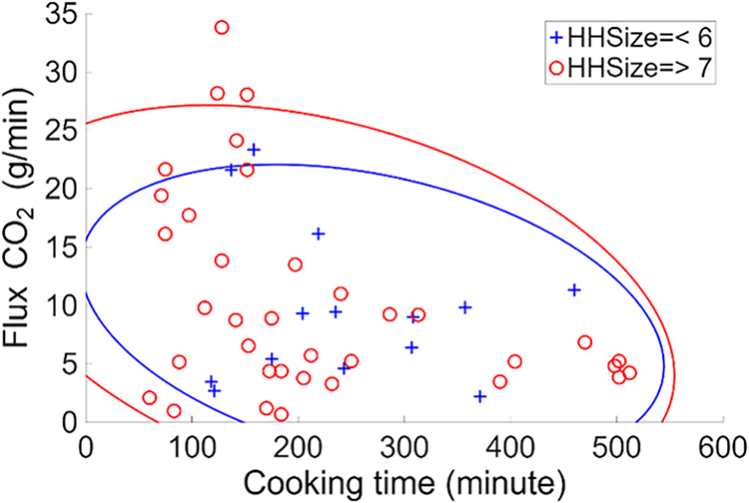
Scatter plot showing CO_2_ flux (g/min) over cooking time (minutes) in relation to household size, a crucial factor in understanding the impact of cooking on indoor air quality and health.

The average upward velocity of the hot air plume rising from the cookstove to the sensor was in the range of 0.7 to 4.1 m/s. This thermal buoyancy can be affected by fuel type, the openness of the kitchen, stove temperature, and ambient conditions of temperature, RH, and pressure. The ANOVA analysis indicated that the stove temperature was the most significant factor (*F*-statistic = 81.93; ρ-value = 2.01 × 10^–11^). Other features, such as fuel type (*F*-statistic = 1.2; ρ-value = 0.32) and kitchen openness (*F*-statistic = 0.7; ρ-value = 0.5), are not statistically significant (Figure SI 8). Ambient environmental conditions had minimal influence.

Various solid fuels used during cooking resulted in different emission trends. For example, charcoal increased the average temperature during cooking (Figure 3). In contrast, animal dung yielded a lower temperature. Although the differences were insignificant, this trend contributed to a higher upward velocity of the rising plume when using charcoal. Based on the BSEC library of BME688 sensor measurements, the average IAQ index indicated that the air quality in the kitchen during cooking was notably poor (Figure 3). Notably, the study found that the extent of openness in the kitchen space, and thus the effectiveness of kitchen ventilation, significantly influenced air quality, demonstrating a practical solution for improving indoor air quality (Figure 4). The results from aerosol sampling by the cascade impactor were compared with SPS30 sensor response (Figure SI 9), which showed a strong positive correlation (*R* ^2^ = 0.747, ρ-value = 1.13 × 10^11^), indicating that the SPS30 sensor can capture trends in particulate emissions relative to the reference method.

**Figure 3:**
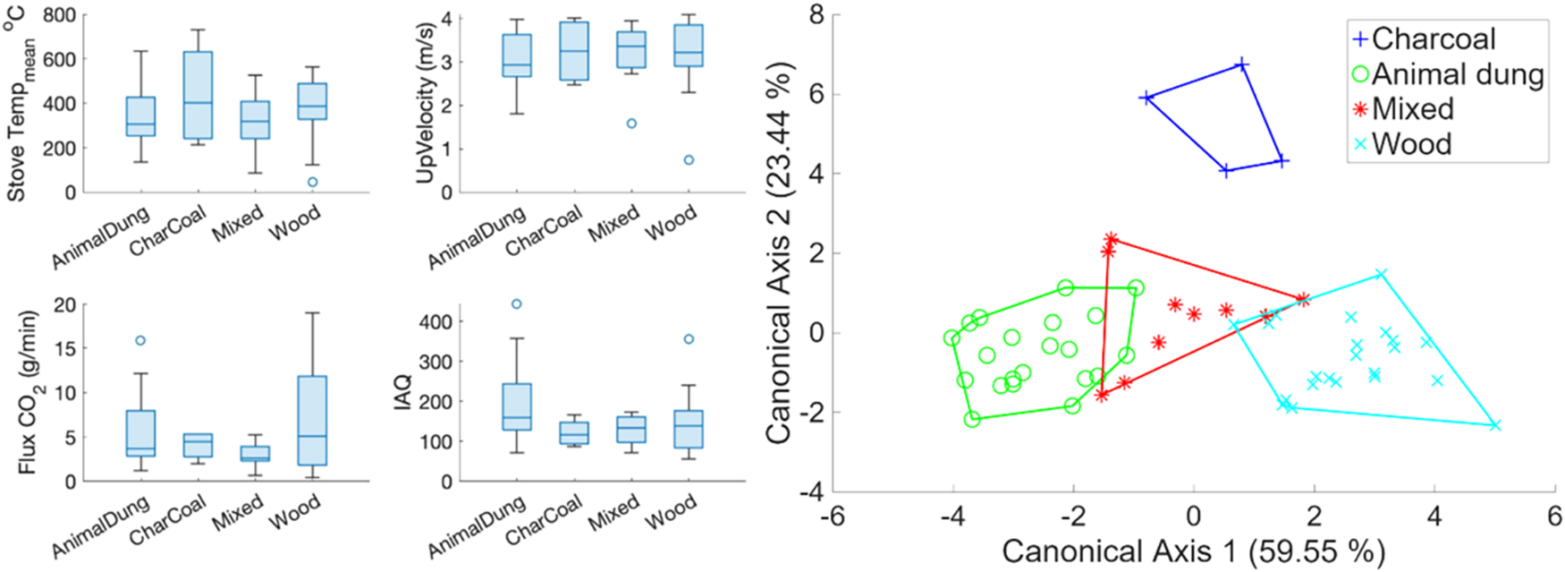
(A) Fuel categories of cooking features, e.g., cookstove mean temperature (°C), upward rising velocity of the plume (m/s), CO_2_ flux (g/min), IAQ mean, and emissions from the cooking events. (B) The LDA score of fuel categories from these cooking-defined parameters subtracts background information.

**Figure 4:**
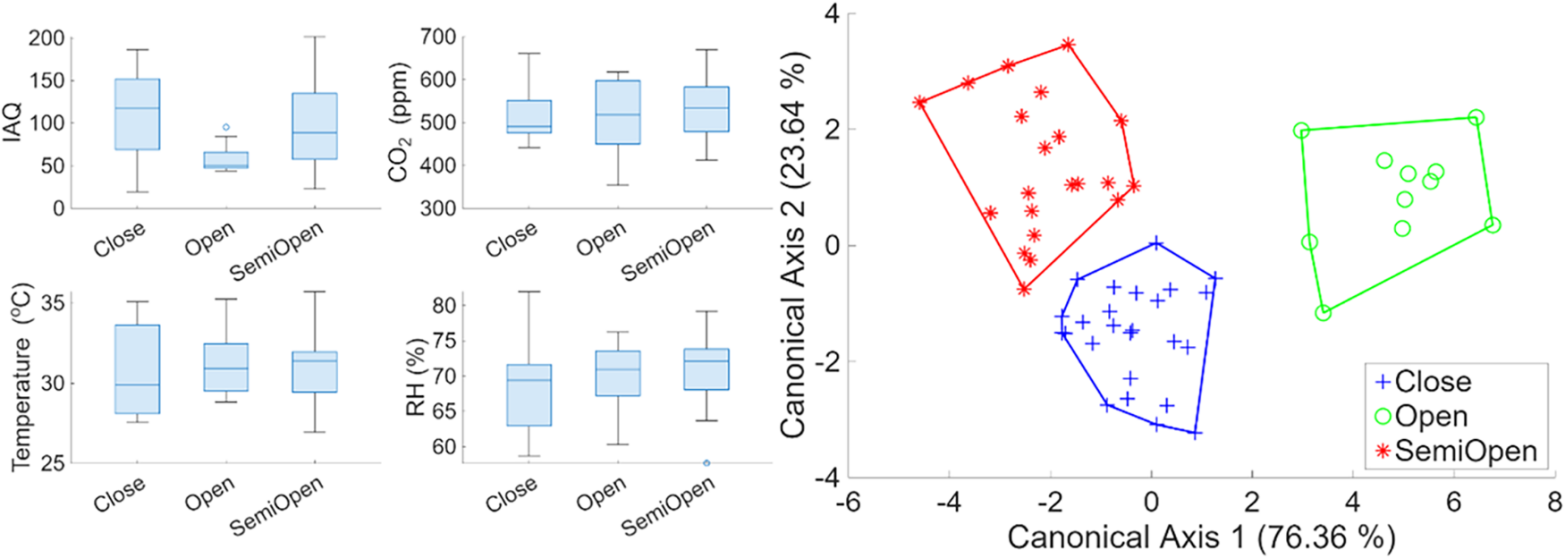
Background environments in rural kitchens are broadly categorized as open, semiopen, and closed ventilation. (A) IAQ, CO_2_ concentration (ppm), and kitchen environment factors, such as temperature (°C) and RH (%). (B) The LDA score of kitchen openness is based on these features.

The sensor-based measurements, which adhered to the factory calibration range during noncooking events, provided a reliable baseline, highlighting the role of advanced sensor technology in ensuring accurate and dependable measurements (Figure 4). The classification study applied to our data set showed that the background environment can be classified based on whether the kitchen is open or closed. As expected, kitchens with better ventilation have better air quality. On the other hand, the CO_2_ concentration closely followed the atmospheric levels and did not show a distinction regardless of the kitchen’s openness. The environment contributed to the background CO_2_ levels observed, which may or may not involve “noncooking” activities such as drying animal dung or warming food and beverages.

Gas resistance values on the BME688 sensors were visually checked for oversaturation of resistance value and distortion of the temperature profile using DAV3E (see example in Figure SI 8). Neither the standard BSEC configuration nor the gas scanner function displayed oversaturation during the cooking event. We performed a *t* test using MATLAB, which revealed no statistically significant differences between the gas resistance values from the BSEC configuration and the gas scanner function. However, the statistical moments (measures of the distribution shape) were significantly different (*h* = 1, ρ-value <0.05). This suggests that the statistical parameters can provide more insights into the distribution of the gas resistance values during measurement.

The linear discriminant analysis results from 142 statistical features showed no classification on the score plot. Hence, we performed a dimensionality reduction through the program’s selection features. We used the Minimum Redundancy and Maximum Relevance (MRMR) algorithm in MATLAB to identify and rank the most important features for classification tasks. This algorithm enabled us to select the most informative features while eliminating redundant or irrelevant ones. Features were systematically removed stepwise, starting with the lowest-ranked ones. This process resulted in 35 key features, including the average slope, median, and FFT for each temperature segment, which produced a distinct classification scheme (Figure 5).

**Figure 5:**
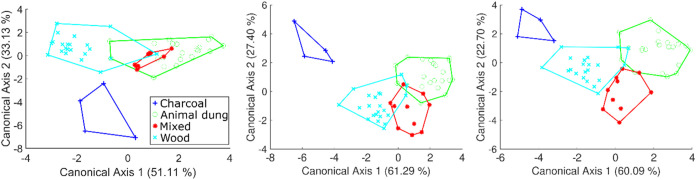
LDA score of BME688 data (A) BSEC, (B) gas scanner function, and (C) gas scanner subtracted from BSEC.

Using the features derived from the BSEC library, LDA could distinguish emissions derived from animal dung, fuelwood, or charcoal but not from a mixture of animal dung, crop residues, and fuelwood. This mixed category could be distinguished from the LDA by leveraging features extracted from the gas scanner data. Given that MOS sensors are affected by changes in temperature and RH,^38,39^ we used features from the BSEC library as a reference to account for these effects. The gas scanner features were adjusted by subtracting them from the corresponding BSEC output was as follows:

8 $${\mathrm{Feature}}_{\mathrm{GasScanSubBsec}}={\mathrm{Feature}}_{\mathrm{BME}0x77}-{\mathrm{Feature}}_{\mathrm{BME}0x76}$$

The temperature and RH influences were effectively corrected to ensure accurate measurements. This adjustment allowed the LDA to achieve better separation of the mixed solid fuel category. We calculated the classification accuracy using MATLAB’s confusionmat function. The results showed that the classification accuracy improved from 88% to 91% when LDA changed from using the BSEC data set to the gas scanner function. The classification accuracy reached 95% after removing the effects of temperature and RH. The temperature cycle on the BME688 enhanced its selectivity in detecting VOC and VOS from emissions.

The cross-sensor comparison helped us detect the sensor issues using a heat map, as shown in Figure SI 7. In cross-sensor comparison during the field campaign, all sensor nodes consistently reported the same low level within an interquartile range. This cross-sensor comparison also refreshed the sensors, allowing them to operate under clean and mildly humid conditions. However, due to limitations in accessing a clean air source and a precise flow meter, we could not perform zero-calibration, where all gas concentrations fall below the sensors’ detection limit, or ensure consistent gas concentrations across different cross-calibration experiments.

## Discussion

We have leveraged advances in microchip, sensor, and battery technology, along with open-source software code for data handling and interpretation. This has enabled our newly engineered sensor array to deliver rapid, high-quality data on biomass-derived emissions in regions where this practice is common. The 32-bit SAMD21 processor efficiently manages and controls the sensors, stores data, and performs statistical calculations, supporting correlations with environmental, health, and remediation efforts. While the sensors require regular calibration, they do not rely on extensive laboratory support or maintenance. They are easy to deploy in the field and very effective for collecting emissions data. The sensor array offers several cutting-edge advantages, including the potential to inform policy decisions and guide public health interventions.

### Monitoring Fuel Emissions

Fuel emission is usually represented as an emission ratio or an emission factor.^14^ While some approaches rely on measuring the mass of dry fuel consumed or laboratory sampling with reference equipment,^40,41^ other studies report emission factors from real cooking environments using carbon mass balance method.^42–45^ This latter method better represents actual conditions, including ventilation, kitchen size, and cooking practices, but require access to costly and highly specialized reference equipment for measuring gaseous and particle phase species emissions. Additionally, it provides a comprehensive assessment by accounting for all carbon forms. However, it has limitations, such as the complexity of accurately measuring all carbon forms in variable field conditions and potential biases if not all carbon is accounted for, which can affect the accuracy of emission estimates due to variations in fuel type or combustion efficiency.^46^ The new sensor array introduced in this study can obtain simultaneous measurements of 230 raw parameters, with data logged at high frequency (every 700 ms for nondefined parameters) in any kitchen environment, providing unparalleled real-time data generation capacity. Fuel emissions monitoring is characterized by either defined parameters using factory-calibrated sensors (60 parameters) or nondefined parameters using a set of BME688 sensors (170 parameters). Collectively, these parameters form a fingerprint that characterizes the kitchen environment, the types of fuel used for cooking, and cooking practices, such as frequency and duration of cooking, representing a detailed air quality picture of the monitored environment. Our direct measurement provides a cost-efficient, faster, and more effective way to achieve this information. This data can then be correlated with lung functionality and cardio-respiratory parameters to offer comprehensive health insights and support the implementation of effective mitigation strategies.

### Dual BME688 Sensor Application

Studies use low-cost VOC sensors but only use the BSEC library for the total VOC measurements.^47^ Using preset libraries, the gas resistance data from these sensors typically provides a summed signal, referred to as total VOC (T-VOC) concentration. However, this provides an incomplete and oversimplified picture of the actual environment. In this study, we used the BSEC gas resistance values as a reference signal to improve VOC characterization while accounting for environmental, temperature, and RH interferences. In parallel, the gas resistance values obtained using the gas scanner function selectively measured compounds with higher affinity to the sensor surfaces at the given temperature. By subtracting the reference signal, a more comprehensive fingerprint of VOC signals can be achieved in the fuel emissions.

### CO_2_ Emissions and Implications

The CO_2_ concentrations observed in our study align with the range and peak shape reported in other studies using reference equipment.^48,49^ In this study, to quantify the output, concentrations measured during noncooking events were subtracted from those during cooking to (1) eliminate drift in the sensor baseline, and (2) account for background environmental differences between kitchens. Unlike the conventional method, in which the emission is based on the amount of fuel consumed and corresponding emission factors from the equilibrium state, CO_2_ emissions in this study are integrated from measurements of CO_2_ in the plume directly above the cookstove during a cooking event. The plume rising from the cookstove during cooking and temperatures exceeding 100 °C diffuse into the ambient kitchen temperature of around 30 °C at an average upward velocity of ∼3.2 m/s.^43,44^ As a result, the air above the cookstove, reaching the sensor, will be quickly refreshed. The result from ANOVA confirms that the thermal gradient from the cookstove is the primary driver of buoyant flow and validates the physical basis of the flux estimation method. The variation in CO_2_ concentration mirrored the fluctuations in stove temperature (Figure SI 10), indicating that the emitted CO_2_ is proportional to the combustion conditions inside the cookstove. For instance, a small amount of fuel is burned at the start of a cooking event, which results in lower temperatures, lower upward velocity, and less CO_2_ emissions. As more fuel burns, the temperature increases, leading to higher upward velocity and higher CO_2_ emissions. Eventually, the temperature drops as the fuel runs out, leading to a lower upward velocity and decreased CO_2_ emissions. Data from 36 households representing varying numbers of subjects indicated that both CO_2_ emission and cooking time were independent of the number of people in a family or the type of solid fuel used for cooking. From our field measurement, the CO_2_ emission was ∼2.3 ± 1.5 kg per cooking event. This aligns with an estimated 2.4 kg CO_2_ emission per cooking event, conventionally derived from an emission factor of 1.6 kg CO_2_ per kg wood and about 1.5 kg wood consumed per cooking event. While several studies have measured and reported a CO_2_ emission factor of roughly 1550–1660 g CO_2_ per kg of firewood or crop residue burned in^14^ cookstoves under control conditions,^14,41^ there are limited studies that measure emissions directly from cookstoves during normal usage in uncontrolled conditions^50,51^ typical of rural households in the Indo-Gangetic Plains. In real-world scenarios, CO_2_ emission factors vary by fuel type and stove design. For instance, an approximate emission factor of 967–1092 g CO_2_ per kg of dry fuel (using a mix of fuels such as wood and animal dung) with an emission rate of ∼25.7 g per minute was reported by Weltman and others in Haryana, India.^50^ Higher values, ranging from 1180–1440 g per kg, were observed for specific fuel-stove combinations, such as brushwood-chulha, in the same region.^42^ Additional studies for traditional chulas in India and Nepal suggested emission factors ranging from 832–1460 g per kg dry fuel, with lower values for dung-based fuels and higher values for wood or crop residue.^43,52^ This estimation is within the CO_2_ flux range noted in our study, from 1.2–33.8 g per minute. This difference could be explained by the type of stove, fuel, cooking habits, and the algorithm used to estimate CO_2_ emissions. In conventional methods, the emission rate calculated uses the CO_2_ concentration at equilibrium during combustion. In contrast, our estimation considers the entire cooking event, from ignition to the final combustion stage. This approach accounts for emissions from the flaming and smoldering phases, providing a more accurate assessment of total emissions during the cooking event.

Extrapolating from the 909 million people living in rural areas in India, this corresponds to around 185 million families.^53,54^ It is reported that about 72.5% of people in rural areas utilize solid biomass for cooking,^2,55,56^ with at least two cooking events per day. This results in estimated emissions ranging from 0.22 to 1.02 Tg of CO_2_ per day (approximately 78.5–373 million tons CO_2_ annually). The US EPA reports estimated 2–6 tons of CO_2_ annually for each cookstove,^38^ which translates to 0.0055–0.0164 tons per day per cookstove. Scaling this to 185 million cookstoves, the total emissions would be approximately 0.83–2.48 Tg of CO_2_ per day. Comparing this with the EDGARv8.0 inventory (155 Tg per year, 0.42 Tg per day), our field-based method provides a realistic lower-bound estimate for emissions from biomass cooking in rural households. Considering the broader context of 3 billion people in low- and middle-income countries, the inefficient combustion of solid fuels and unprocessed biomass highlights the urgent need for a comprehensive evaluation to assess significant societal health and environmental risks associated with using solid biomass for cooking.^2^ It also underscores the importance of investing in alternative cooking technologies and better energy resources, as well as promoting behavioral changes to mitigate these risks and improve public health outcomes.

Many researchers in low-resource settings need devices like ours that effectively monitor rural environments. These devices must measure both high and low emission levels, be easy to clean and maintain, and remain reliable even without a stable power supply. Access to such low-cost devices is crucial for many study groups because it enables them to collect multiple measurements over extended periods in each household. Deploying sensor arrays like these allows a comprehensive temporal and spatial monitoring protocol, facilitating the creation of a robust database for tracking emissions and understanding their environmental and health impacts over time and across different locations. Moreover, integrating commercial low-cost sensors into an appropriate, cost-effective design and proper data handling capacity (as demonstrated in this study) can provide valuable scientific insights. This transformative approach will enhance our ability to assess the impacts of household energy consumption more thoroughly. Moreover, transitioning to robust and precise sensing systems can significantly improve our understanding of stove use, pollutant concentrations in kitchens and living areas, and personal exposures among residents. It also aids in identifying emerging issues, supports community engagement in discussing local pollution sources, and aids in implementing remedial measures from individual households to the community level.

## Supplementary Material



## Notes

Additional figures and photographs detailing the sensor node design, sensor evaluation, cross-sensor check experiment setup, field measurement results, and a bill of materials for constructing the sensor node (PDF)

## Abbreviations

VOC: volatile organic compounds
MOS: metal oxide semiconductor
IAQ: indoor air quality
BSEC: Bosch Sensortec Environmental Cluster
PM: particulate matter
