# Supplementary material for: Tagging Emissions from Indoor Biomass Combustion with a Cost-Effective Sensor Array: From Design to Field Deployment in Rural Indian Households
Source: Environ Sci Technol. 2025 Jul 17;59(30):15730–40. doi: 10.1021/acs.est.4c08533 (PMC12329721; doi:10.1021/acs.est.4c08533)
Supplement: Supplementary file 1 [file es4c08533_si_001.pdf]

Supporting information

Tagging Emissions from Indoor Biomass Combustion with a Cost-Effective Sensor Array: From Design to Field Deployment in Rural Indian Households.

Nguyen Thanh Duc[1*], Daniel Montecinos[1], Jay Prakash Kumar[2], Sayantan Sarkar[3], Roshan Wathore[4,5], Johannes Felix Amann[6], Julian Joppich[6], Sumedha Lawande[3], Rajesh Kumar Ranjan[2], Joyanto Routh[1]

[1]Department of Thematic Studies-Environmental Change, Linköping University, Linköping, 58183, Sweden

[2]Department of Environmental Science, Central University of South Bihar, Gaya, 824236, India

[3]School of Civil and Environmental Engineering, Indian Institute of Technology - Mandi, 175005, India

[4]Academy of Scientific and Innovative Research (AcSIR), Ghaziabad, 201002, India

[5]CSIR-National Environmental Engineering Research Institute, CSIR-NEERI, Nagpur, 440020, India

[6]Lab for Measurement Technology, Saarland University, Campus A5 1, 66123 Saarbrücken, Germany

Correspondence to: Nguyen Thanh Duc (thanh.duc.nguyen@liu.se)

This supporting information contains ten pages, twelve figures and one table.

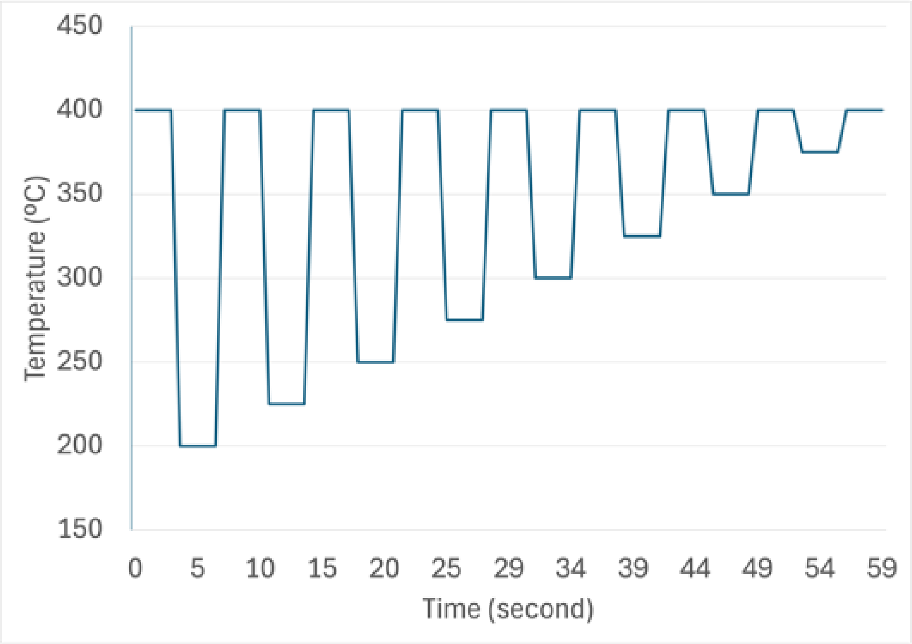

Figure SI 1: Temperature cycle operation of BME688 sensor at I2C address 0x77.

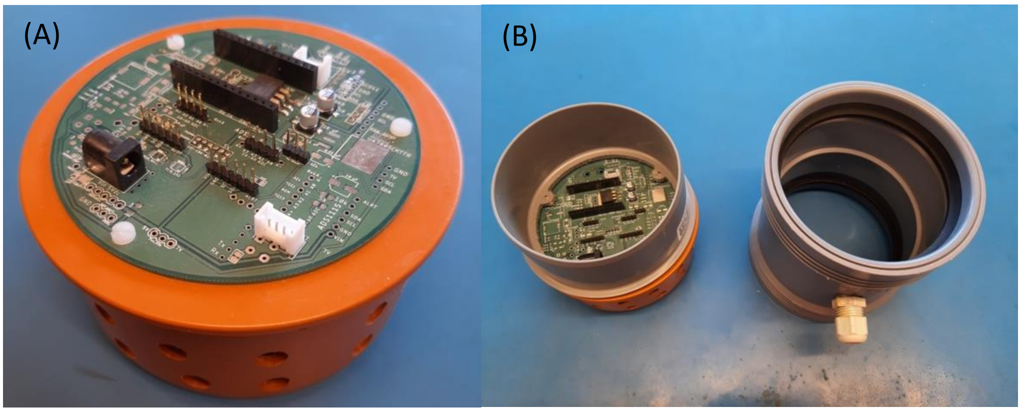

Figure SI 2: (A) Electronic carrier board fit on a sewage pipe end stop. (B) The sensor housing is made from the drainage pipe sliding sleeve (110 mm ID) and the sewage pipe end stop (110 mm ID).

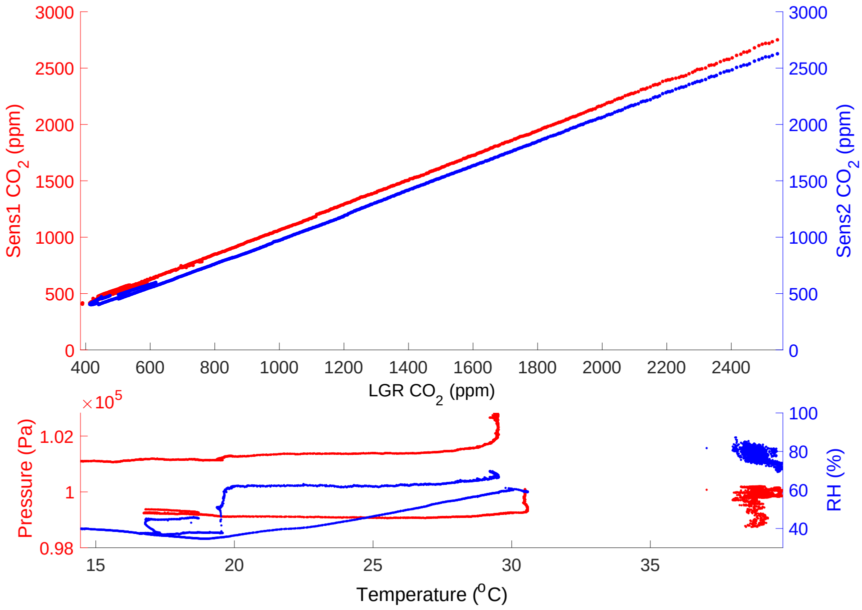

Figure SI 3: This figure showed two CO_2 sensors responses with a Los Gatos Research (LGR) Instrument under varying temperature and RH conditions. Sensor 1 data (Slope = 1.09, R2 = 0.99) is presented in red, and Sensor 2 data (Slope = 1.05, R2 = 0.99) is presented in blue. CO_2 sensors were placed in a closed gas chamber, where the headspace gas was circulated with the LGR instrument. The gas chamber was placed in a water thermal bath for temperature control, and the gas flow was bubbled through water to control RH in the chamber. Approximately 60 mL of 20% CO_2 standard gas were injected into the chamber. The results show good agreement between the CO_2 sensors' responses and the LGR instrument.

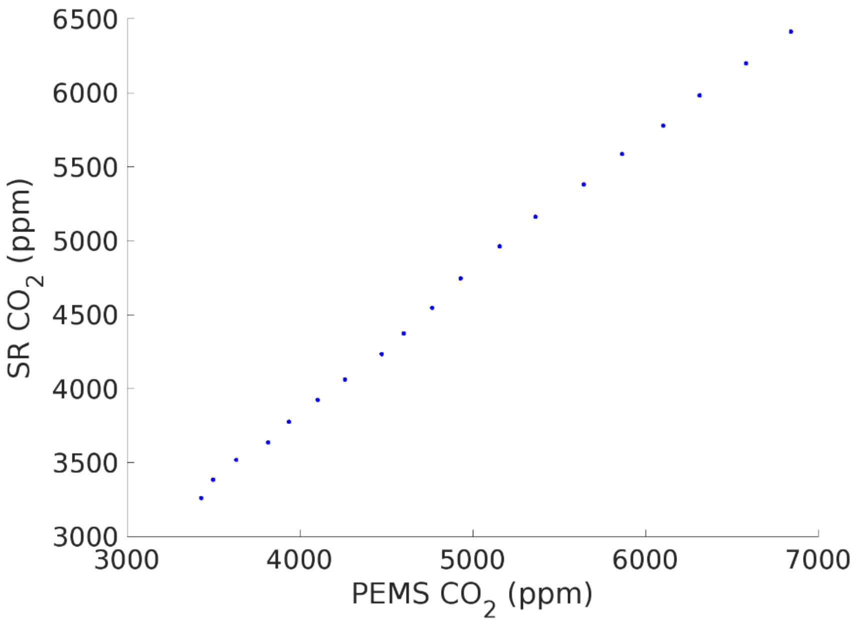

Figure SI 4: This figure illustrates the cross-calibration between a CO_2 sensor and CO_2 measurement of a Portable Emissions Monitoring System (LEMS 3006 by Aprovecho). The calibration was performed during emissions measurements from a cookstove test conducted in CSIR-NEERI laboratory. The results (Slope = 0.98, R2 =0.995) demonstrate good agreement between the two sensors, validating the accuracy and reliability of the measurements.

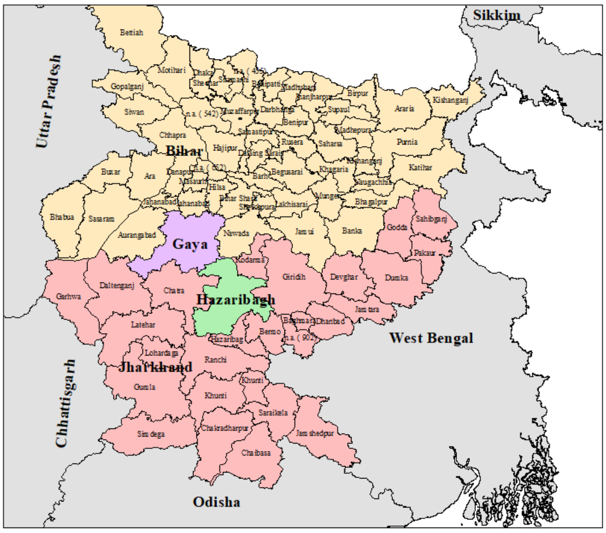

Figure SI 5. Map of investigated site: Gaya and Hazaribagh districts.

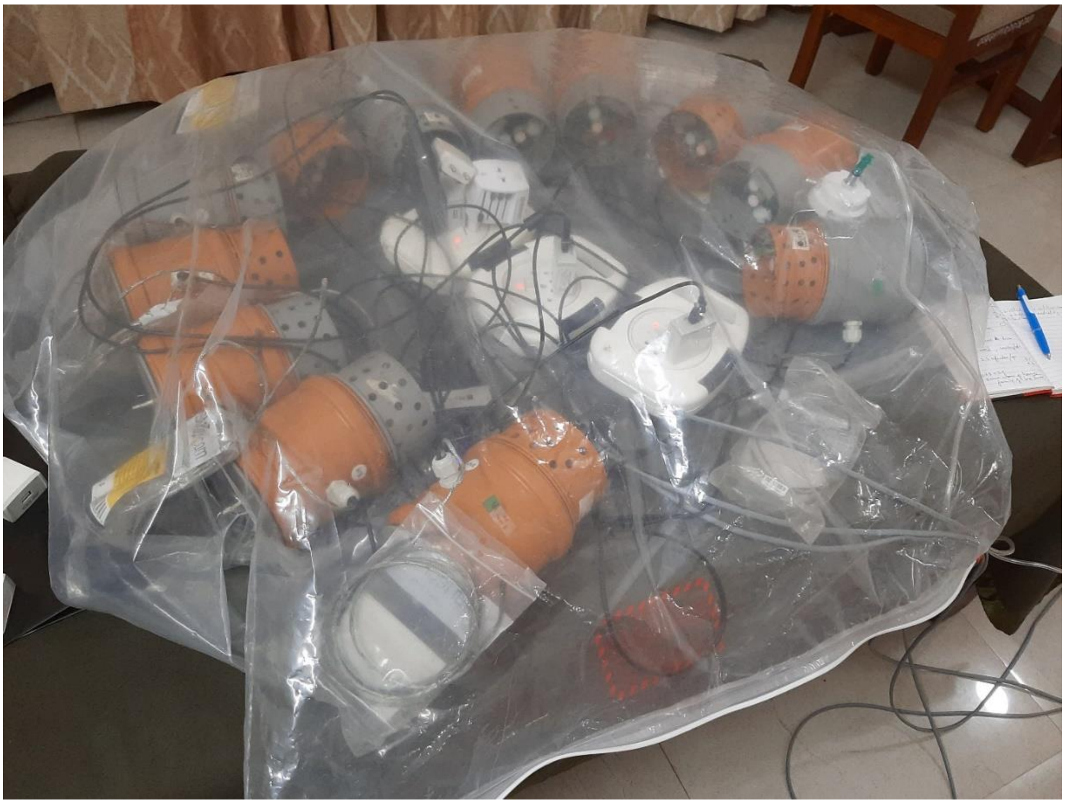

Figure SI 6: Cross calibration setup. This glove bag was filled with medical oxygen (about 2-L per minute) for about 2 hours.

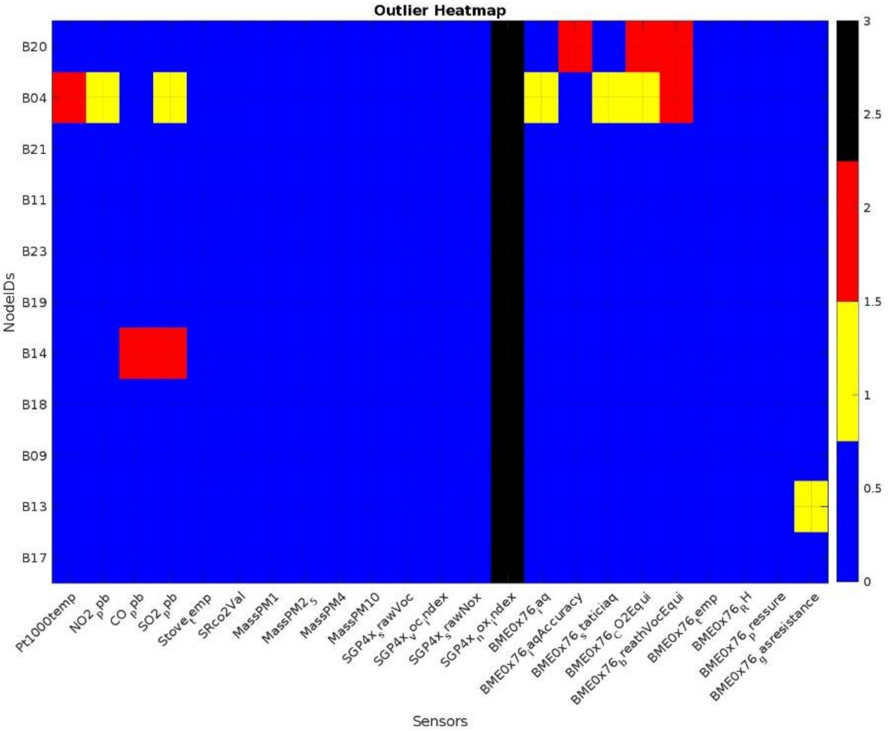

Figure SI 7: Heat map to detect functionality and monitor sensor capacity. Sensor status is interpreted based on colors: Blue: functional; Yellow: sensor drift; Red: sensor problem, needs to be replaced; Black: usually connection/conversion problem.

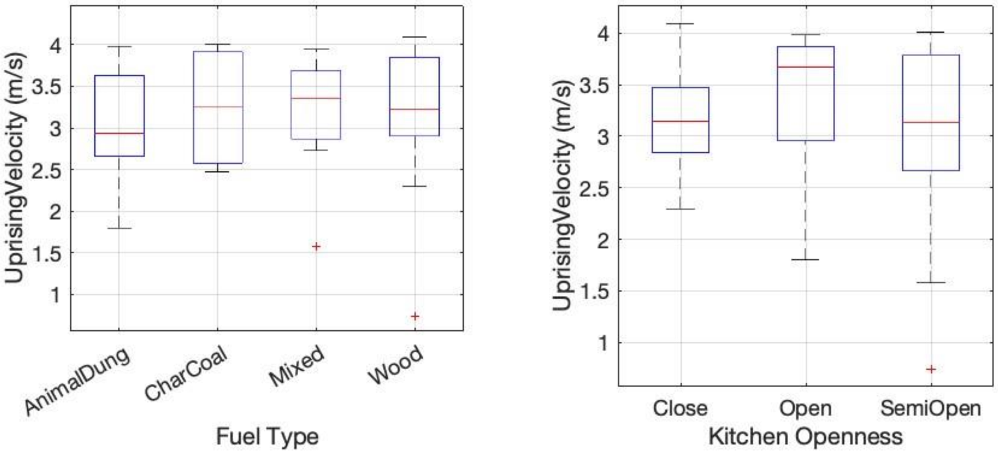

Figure SI 8: Box plots showing the distribution of uprising velocity (m/s) during cooking events, categorized by (left) fuel type—Animal Dung, Char coal, Mixed, and Wood—and (right) kitchen openness—Closed, Semi Open, and Open. Although the differences were not statistically significant, higher uprising velocities were observed for wood and mixed fuels, as well as in more open kitchen settings. These trends suggest greater thermal buoyancy and a potentially enhanced dispersion of pollutants under these conditions.

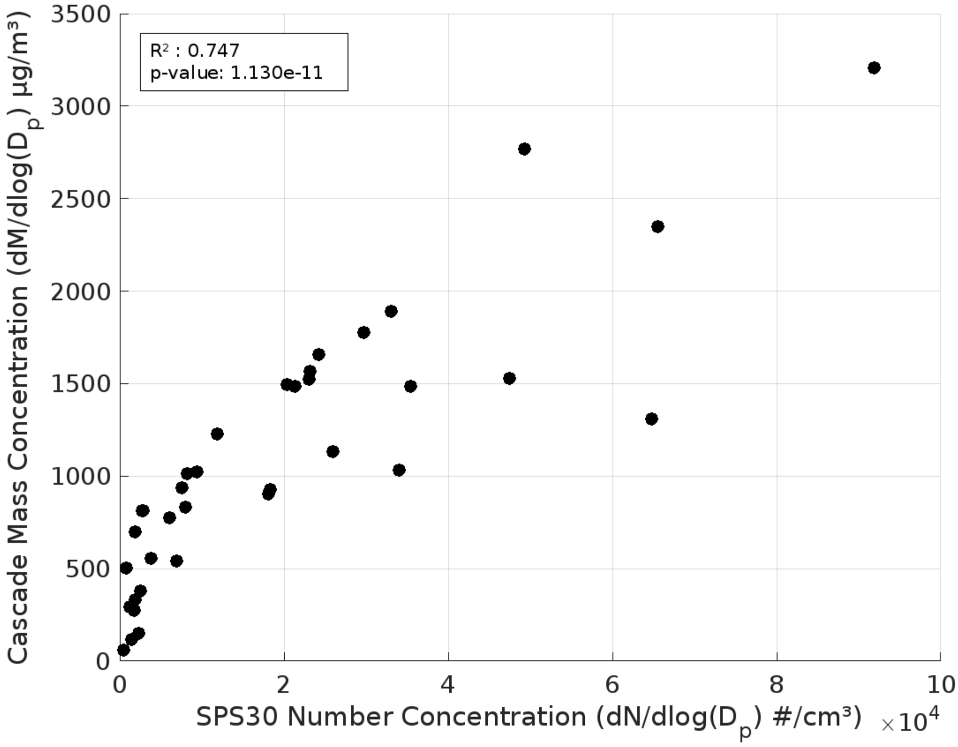

Figure SI 9: Scatter plot showing the correlation between corrected normalized particle number concentration measured by the SPS30 sensor (dN/dlog(D_p), #/cm³) and normalized mass concentration measured by a cascade impactor (dM/dlog(D_p), μg/m³). Each point represents a paired observation during cooking events. The strong positive correlation ($R^2 = 0.747$, ρ - value = 1.13×10^{-11}) indicates that the SPS30 sensor is able to capture trends in particulate emissions relative to the reference method.

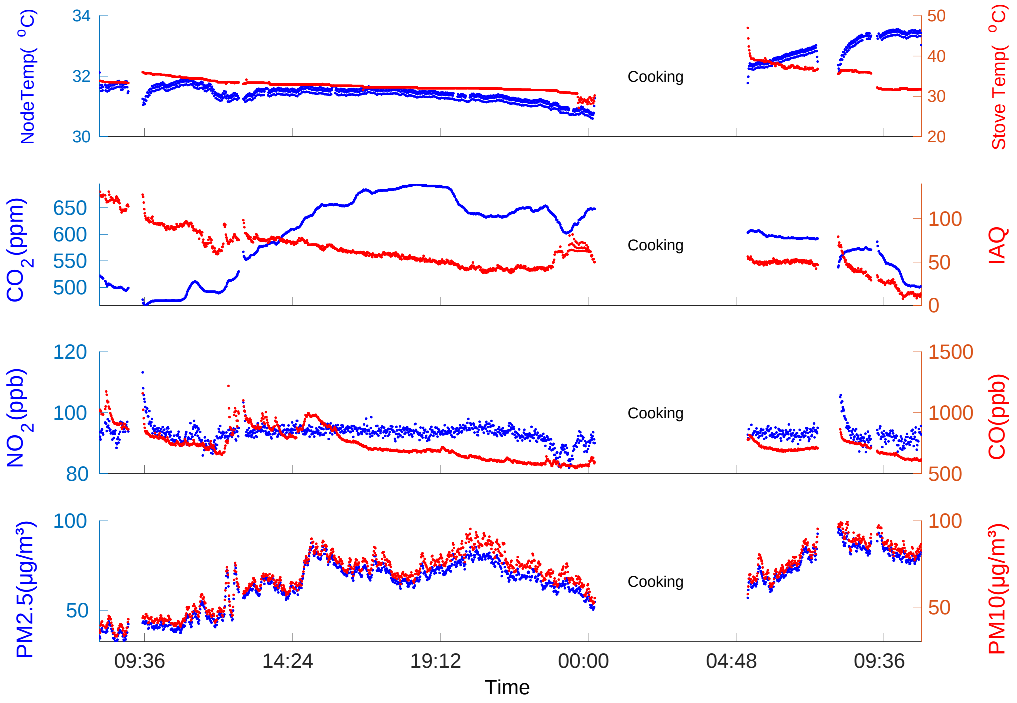

Figure SI 10: And exmaple of a background measurement at one location of the study. The time axis is GMT timezone. There are few data gap due to power cut which can be marked in the data file by checking if GPS_Time value is NaT and the adrupt high value of electrochemical sensor (NO_2, CO and SO_2), these sensors required at least 40 minutes to stabilize. This initial non-stable data were removed.

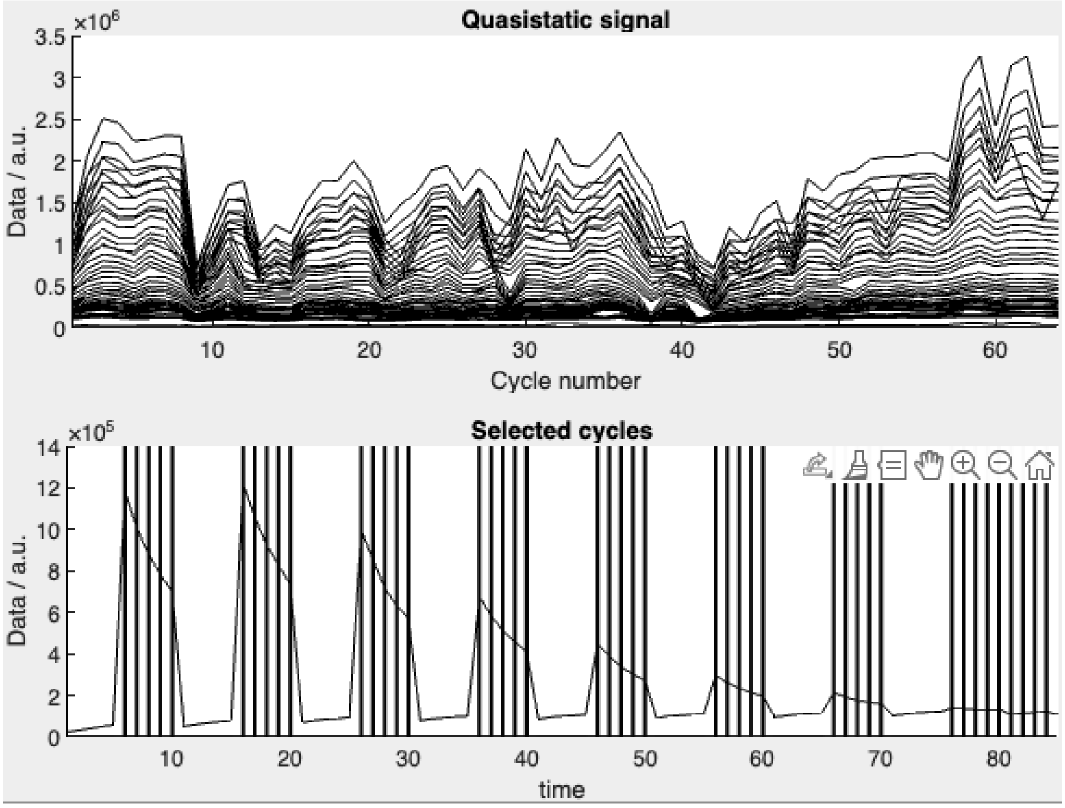

Figure SI 11: Evaluating temperature cycle on BME688 sensor

The feature matrix for BME0x77 of a cooking event can be represented as:

$$Feature_{BME0x77} = \begin{bmatrix} avgSlo\_400 & median\_400 & std\_400 & range\_400 & iqr\_400 & ske\_400 & kur\_400 & fft\_400 \\ avgSlo\_200 & median\_200 & std\_200 & range\_200 & iqr\_200 & ske\_200 & kur\_200 & fft\_200 \\ avgSlo\_400 & median\_400 & std\_400 & range\_400 & iqr\_400 & ske\_400 & kur\_400 & fft\_400 \\ avgSlo\_225 & median\_225 & std\_225 & range\_225 & iqr\_225 & ske\_225 & kur\_225 & fft\_225 \\ \vdots & \vdots & \vdots & \vdots & \vdots & & \vdots\ \vdots\ \vdots & \\ avgSlo\_400 & median\_400 & std\_400 & range\_400 & iqr\_400 & ske\_400 & kur\_400 & fft\_400 \end{bmatrix}$$

(Equation SI 1)

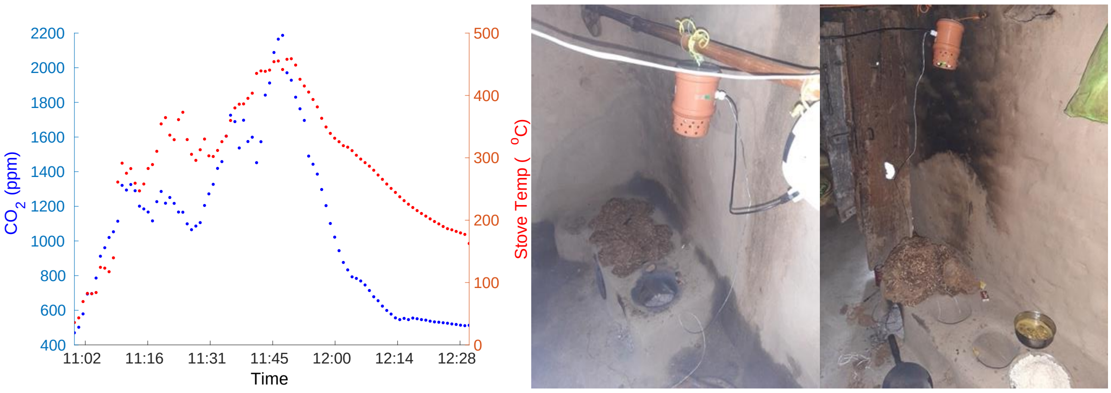

Figure SI 12: Demonstration of the correlation between CO_2 concentration variations and stove temperature, along with the deployment of sensor nodes directly above the stove.

Matlab datahandling steps in Process_CookstoveData.m:

1) Manual: Declare Location ID (field site ID), Node ID (which sensor node is used), and Filenametoprocess (in format LocID_NodeID_DateofDatacollection)
2) Automatic: All raw data in csv format is merged (if the deployment is more than a day) and imported.
3) Automatic: The NO_2, CO, SO_2 concentration is calculated according to the calibration documents provided by Alphasense.
4) Automatic: Extract and remove the cross-calibration data from the data table.
5) Automatic: Cleanup the data table.
6) Automatic: Perform K-means clustering to find out the dominant background data.
7) Automatic: Determine the cooking event from the background data.
8) Automatic: Plot and extract the background parameters from non-cooking period.
9) Manual: select and assign the cook data into an individual data table.
10) Automatic: Extract cooking parameters from the cooking data table.

Table SI 1: Bill of materials

	**Items**	**Description**	**MFG Part Number**	**Manufacturer**	**Amount per board**	**Price per sensor node (SEK)**
Solder on PCB	Printed Circuit Board				1	142
	10 µF	WCAP-AS5H_6.3X5.5(DXL)	865230643009	Wurth Elektronik	2	2,98
	REG1117F-3.3KTTT	TO254P1524X483-4N	REG1117F-3.3/500	Texas Instruments	1	28,781
	BME connector	2.54mm female header H8.4mm 1x7p	RND 205-00647	RND	1	1,88
	Sunrise connector	2.54mm female header H8.4mm 1x4p	RND 205-00644	RND	1	1,1013
	Sunrise connector	2.54mm female header H8.4mm 1x5p	RND 205-00645	RND	1	1,1913
	ADS115 connector	2.54mm female header H8.4mm 1x6p: 2 pcs	RND 205-00646	RND	3	6
	Arduino connectors	2.54mm female header H8.4mm 1x14p: 2pcs	61301411821 WR-PHD	RND	2	12,2842
	SPS30 connector	ZH 1.5mm header top entry 5way	B5B-ZR (LF)(SN)	JST	1	1,23
	Power supply socket	WR-DC Right Angle DC Socket Rated At 5.0A, 24.0 V, Panel Mount, length 9.0mm, Gold, Tin	694106301002	Wurth Elektronik	1	9,81
	Break away	(2x3pins=6pins) PRT-00116 come with lots of pins	M20-9990345	HARWIN	1	3,24
	2w jumpers 2.54mm				1	8,2
Top parts	Arduino MKR GPS		ASX00017	Arduino	1	323,93
	CR1216 battery for MKR GPS 25 mA				1	25,1
	MKR Zero		ABX00012	Arduino	1	283
	ADS 1115		41020542	Adafruit	2	336

	Wire connector SPS30	ZH-2.5mm receptacle housing 5w	ZHR-5	JST	2	1,396
	Wire for SPS30	JST Female SZH to Female SZH Crimped Wire, 150mm, 0.05mm²,	01SZHSZH-30L-150	JST	5	12,45
	SD memory card	Memory Card, microSD, 16GB, 60MB/s, 30MB/s, Black / Blue	NT02P500STN-016G-R	Netac	1	25,1
	Power supply transformer 5V1A		1001-0108-520	Ansmann	1	54,15
	USB 2.0 Cable, Male USB A to Male 2.1mm DC Power Cable, 2m		USB2TYPEM2M	StarTech.com		61
	Housing, weather protection	drainage pipe sliding sleeve (110 mm ID) and the sewage pipe end stop (110 mm ID)			1	200
Bottom Sensor	BME688 -VOC, VOS, H2, Temp, RH, P.		5046	Bosch	2	365,87
	SGP40 Qwiic Air Quality Sensor, SparkFun Electronics		SEN-18345		1	
	SPS30 - PM sensor PM1, PM2.5, PM 4, PM 10	Sensirion SPS30, Air Quality Sensor for Air Quality Monitors	SPS30	Sensirion	1	383
	SenseAir Sunrise - CO2 sensor			SenseAir	1	360
	Sensor carrier	3-way + PID AFE: (NO2/O3) (CO/SO2/H2S) (CO/SO2/H2S) + PID for 4-pin A Series Sensors		Alphasense	1	2096
	SO2	Sulfur Dioxide (SO2) Sensor - A-Series -	SO2-A4F	Alphasense	1	640
	NO2	Nitrogen Dioxide (NO2) Sensor - A	NO2-A43F	Alphasense	1	524
	CO	Carbon Monoxide (CO) Sensor - A-Series - 4-pin PPB	CO-A4F	Alphasense	1	712
	Total					**6622**
